# Twenty Four‐Hour Movement Behaviours Research Among Australian Children and Adolescents: A Scoping Review

**DOI:** 10.1002/hpja.70021

**Published:** 2025-02-19

**Authors:** Mosharop Hossian, Mehwish Nisar, Gregore Iven Mielke, Asaduzzaman Khan

**Affiliations:** ^1^ School of Health and Rehabilitation Sciences The University of Queensland Brisbane Australia; ^2^ School of Public Health The University of Queensland Brisbane Australia

**Keywords:** academic performance, adolescent health, physical activity, screen time, sleep patterns

## Abstract

**Background:**

Adherence to 24‐h movement behaviour (24‐h MB) guidelines, encompassing moderate‐to‐vigorous physical activity (MVPA), recreational screen time (ST) and sleep, is low among Australian children and adolescents, with poorly understood contributing factors. This review synthesised current evidence to identify areas requiring further exploration in this demographic.

**Methods:**

A systematic search in PubMed, Scopus, Web of Science, SportDiscus and CINAHL identified articles published from June 2016 and March 2024. Peer‐reviewed articles in English focusing on healthy school‐aged children and adolescents (5–17 years) addressing 24‐h MB guidelines, including those using compositional data analysis (CoDA), were included.

**Results:**

Twenty‐three articles met the inclusion criteria. Most were of fair quality and used cross‐sectional designs and self‐reported measures. Adherence to 24‐h MB guidelines ranged from 2% to 22% in Australia. The included articles focused on prevalence (*n* = 12), correlates (*n* = 5), health outcomes (*n* = 13), academic performance (*n* = 4), and an intervention (*n* = 1). Factors negatively associated with adherence included lower socioeconomic status, transitions from primary to secondary school, and family history of diabetes. Positive outcomes associated with adherence included improved academic performance, quality of life, and physical and mental health. Important research gaps were identified, particularly regarding intervention studies and limited exploration of a causal relationship between adherence, contributing factors, and related outcomes. Compositional analyses (*n* = 10) suggested that reallocating time from sedentary behaviour to MVPA may improve fitness and academic performance, whereas increasing sedentary time may lower sleep efficiency.

**Conclusions:**

A comprehensive understanding of factors associated with adherence to 24‐h MB guidelines among Australian children and adolescents is lacking. More longitudinal and interventional studies using objective measures are needed to establish causality and provide a deeper understanding of adherence to 24‐h MB guidelines among children and adolescents in Australia.

**So What:**

This scoping review underscores the need for more longitudinal and intervention‐based research to explore causal relationships between adherence to 24‐h MB guidelines and potential benefits for health, academic performance, and quality of life in Australian children and adolescents.

## 1 Introduction

The 24‐h movement behaviours (24‐h MB) guidelines emphasise integrating physical activity (PA), adequate sleep and limited sedentary behaviour (SB), particularly screen time (ST) into the daily lives of children and adolescents [1, 2]. The rationale for their integration is supported by the systems theory, which posits that the components of a system are more effective together than in isolation [3]. This integration aims to promote overall well‐being, with each behaviour contributing in a complementary way to children and adolescents' health and development. Each of these behaviours may serve a specific function; PA may enhance physical self‐perception [4], sufficient sleep could be essential for cognitive and emotional growth [5] and controlled ST may promote a healthier lifestyle balance [6]. By addressing these key components holistically, this integrated approach may enhance the overall development by simultaneously promoting physical health [7, 8], cognitive abilities [9] and emotional well‐being [10]. Moreover, adhering to these guidelines may foster important life skills such as discipline and time management, beneficial in both educational and social contexts [11, 12].

Initially introduced in Canada [2], the guidelines were later adapted in Australia [1] to specifically address the health and developmental needs of Australian children and adolescents, such as rising levels of childhood obesity [13], mental health issues [14] and sedentary lifestyles [15] among the Australian children and adolescents. Additionally, factors such as the increasing prevalence of digital media [16], diverse geographical distribution [17] and varying socioeconomic status across the country [18] necessitate such a tailored approach. Building on the foundational principles of the 24‐h MB guidelines, specific recommendations have been outlined for Australian children and adolescents to encourage a balanced daily routine [1]. These guidelines suggest that school‐aged children (5–17 Years) should engage in at least 60 min of moderate to vigorous physical activity (MVPA) daily, limit recreational ST to fewer than 2 hours and ensure adequate sleep—9–11 h for children aged 5–13 and 8–10 h for adolescents aged 14–17 [1, 2].

**FIGURE 1:**
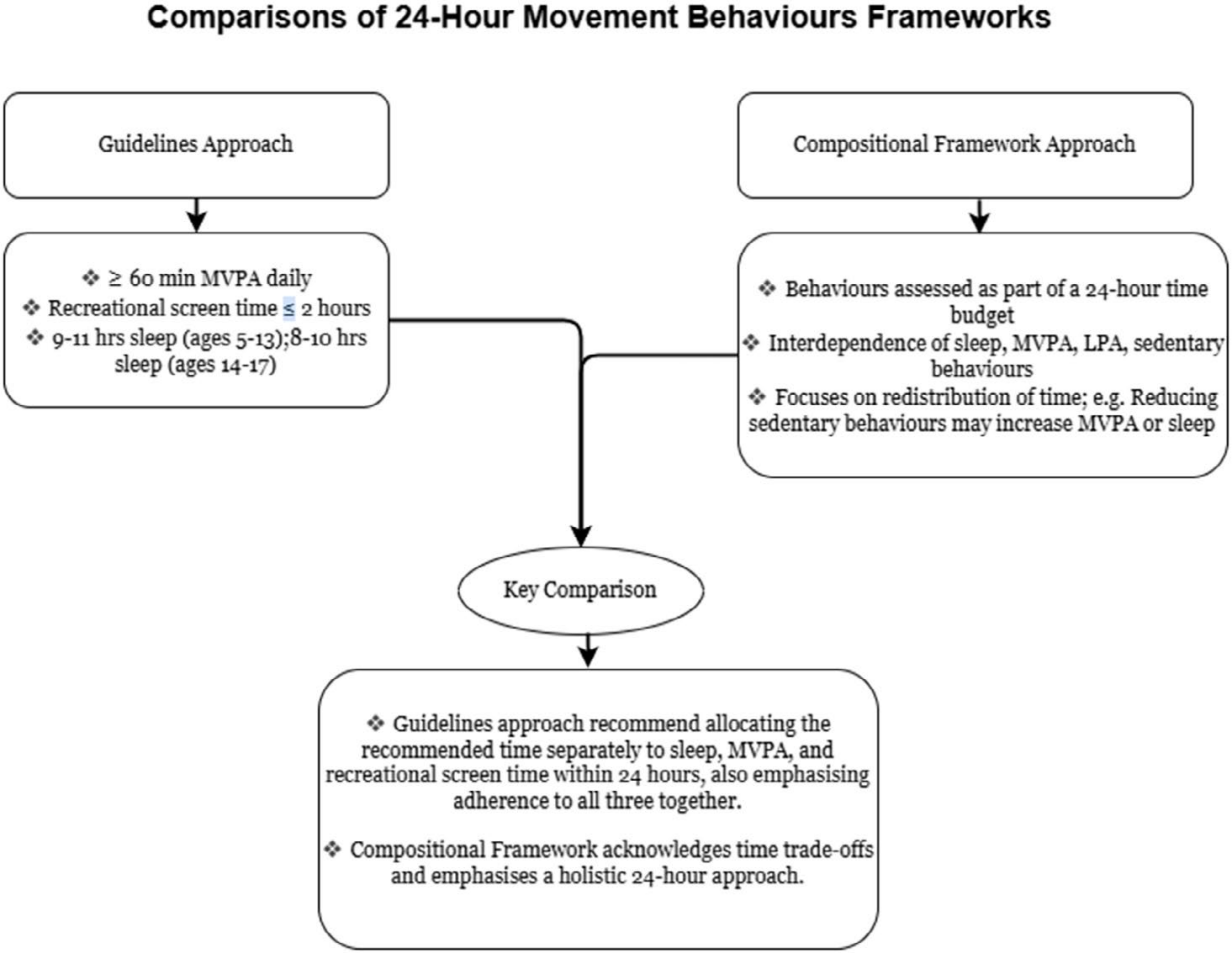
Overview of 24‐hour movement behaviours frameworks.

An alternative perspective to the separate guidelines approach is the compositional framework, which argues that behaviours within a 24‐h day are inherently interdependent, making it artificial to assess them in isolation [19]. From this compositional angle, the focus shifts from individual behaviours to the overall distribution of time across the day, emphasising how changes in one behaviour (e.g., reducing sedentary time) may change others (e.g., increasing sleep or MVPA). This perspective considers the “system” of behaviours as a whole, highlighting how the balance of time use can contribute to multiple health and developmental outcomes simultaneously [3]. For instance, compositional data analysis (CoDA) may provide a detailed understanding of how reallocating time among behaviours (e.g., replacing sedentary time with MVPA) may result in significant improvements in physical, cognitive, and emotional health [20]. Understanding these two conceptual frameworks – the separate guidelines approach and the compositional framework (Figure 1) – is essential for interpreting the existing research and developing interventions. While the former allows targeted strategies to address specific health and developmental outcomes, the latter provides a holistic lens to evaluate the synergistic outcomes of time‐use behaviours.

While several studies have explored integrated 24‐h MB in Australian children and adolescents (5–17 years) [7, 8, 21, 22, 23, 24, 25, 26, 27, 28, 29, 30, 31, 32, 33, 34, 35, 36, 37, 38, 39], the findings remain fragmented and lack a systematic synthesis. This absence of synthesis hinders our understanding of the overall picture of prevalence, determinants, and associations of these behaviours with important outcomes in the Australian context. Although global reviews have provided valuable insights [40, 41, 42], they do not account for the unique environmental, cultural and socioeconomic factors specific to Australia [43, 44]. Given the growing concerns about childhood obesity‐related comorbidities such as dyslipidaemia [45], rising ST [46], and mental health challenges in Australia [47], it is crucial to develop a clearer understanding of the prevalence of compliance with these behaviours, their determinants and their associations with key health outcomes. Moreover, given the two distinct conceptual frameworks – Separate Guidelines and Compositional – underpinning the 24‐h movement behaviours, it is essential to consider both perspectives in the analysis. By analysing the relative distribution of time across behaviours such as MVPA, light physical activity (LPA), sedentary behaviour (SB) and sleep, CoDA provides insights into how shifts between these activities may influence measurable outcomes. This is especially important in understanding which time reallocations (e.g., reducing sedentary time by 30 min to add more MVPA) are associated with the most significant health and developmental improvements for Australian children and adolescents.

To address the gaps, the aim of this scoping review is to synthesise current evidence on 24‐h MB among Australian children and adolescents (ages 5–17). Specifically, the objectives are to (a) map existing study characteristics and report adherence to 24‐h MB guidelines as a population characteristic, (b) better understand contributing factors such as geographic location, socioeconomic status, age group, and gender, (c) synthesise the evidence on the outcomes of adherence to these guidelines, and (d) identify areas requiring further exploration.

## 2 Materials and Methods

### 2.1 Study Design

A comprehensive systematic search was conducted to identify articles on 24‐h MB of Australian children and adolescents (5–17 Years), spanning from June 2016 to March 2024. The start date was chosen as June 2016 aligning with the publication of the Canadian 24‐h movement guidelines [2], which provided the foundational framework for similar guidelines adapted in Australia. This was due to their evidence‐based, holistic approach that combined recommendations for all three key daily movement behaviours. The search used five databases including PubMed, Scopus, Web of Science, SportDiscus and CINAHL to ensure thorough coverage of the subject matter. Concurrently, we reviewed existing systematic reviews to identify additional relevant articles, thereby enhancing the comprehensiveness of our review. A targeted search was also conducted for articles using Compositional Data Analysis (CoDA). This scoping review adhered to the Preferred Reporting Items for Systematic Reviews and Meta‐Analyses for scoping review (PRISMA‐ScR) guidelines [48] and was registered in the PROSPERO database under reference number CRD42024550065 [49], ensuring adherence to established research standards.

### 2.2 Search Terms and Strategies

A structured search strategy focused on 24‐h MB terms was used to identify relevant articles (Data S2). This included terms like “physical activit*”, “screen time”, “sleep*”, and “movement*”, and on guidelines with keywords such as “guideline*” and “recommendation*”. Our demographic and geographic scope targeted “Australia”, along with specific age groups using “child*” and “adolescen*”. The strategy was adapted to suit each database; for instance, in PubMed, we searched within the MeSH and Title/Abstract fields, whereas in Scopus, our search was extended to Title/Abstract/Index terms fields. Additional searches were conducted for Compositional Data Analysis articles using “compositional” and “24 h*”.

### 2.3 Study Selection

We organised the publications retrieved from the selected databases using Covidence and removed the duplicates. The selection criteria were as follows: (1) articles focusing on generally healthy school‐aged children and adolescents (5–17 years), adhering primarily to the 24‐h MB guidelines. Notably, some articles employing CoDA have introduced light physical activity (LPA) as a distinct fourth component. (2) All study designs were considered, except for reviews including systematic review, scoping reviews, narrative reviews, and study protocols. Reviews were excluded to focus our analysis on primary research studies only, thereby avoiding data duplication and ensuring a comprehensive synthesis of original findings (3) Only articles published in English were included in this review. (4) Articles on clinical populations, adults, infants, toddlers, and non‐peer‐reviewed (grey) literature were excluded. Two reviewers (MH and MN) independently reviewed the abstracts and full texts with no discrepancies in the selection of eligible publications.

### 2.4 Data Extraction and Summarisation

Summary tables were prepared to include information on the study type, sample size, data collection methods, age and sex of the participants, year of study, and primary outcomes. Due to the variety of methodologies and measurement tools reported across articles, summarising this information was chosen as the most effective approach to provide a clear overview and identify research gaps. Narrative synthesis methods were employed to identify research gaps, aligning with the scoping review framework.

### 2.5 Quality Assessment

**FIGURE 2:**
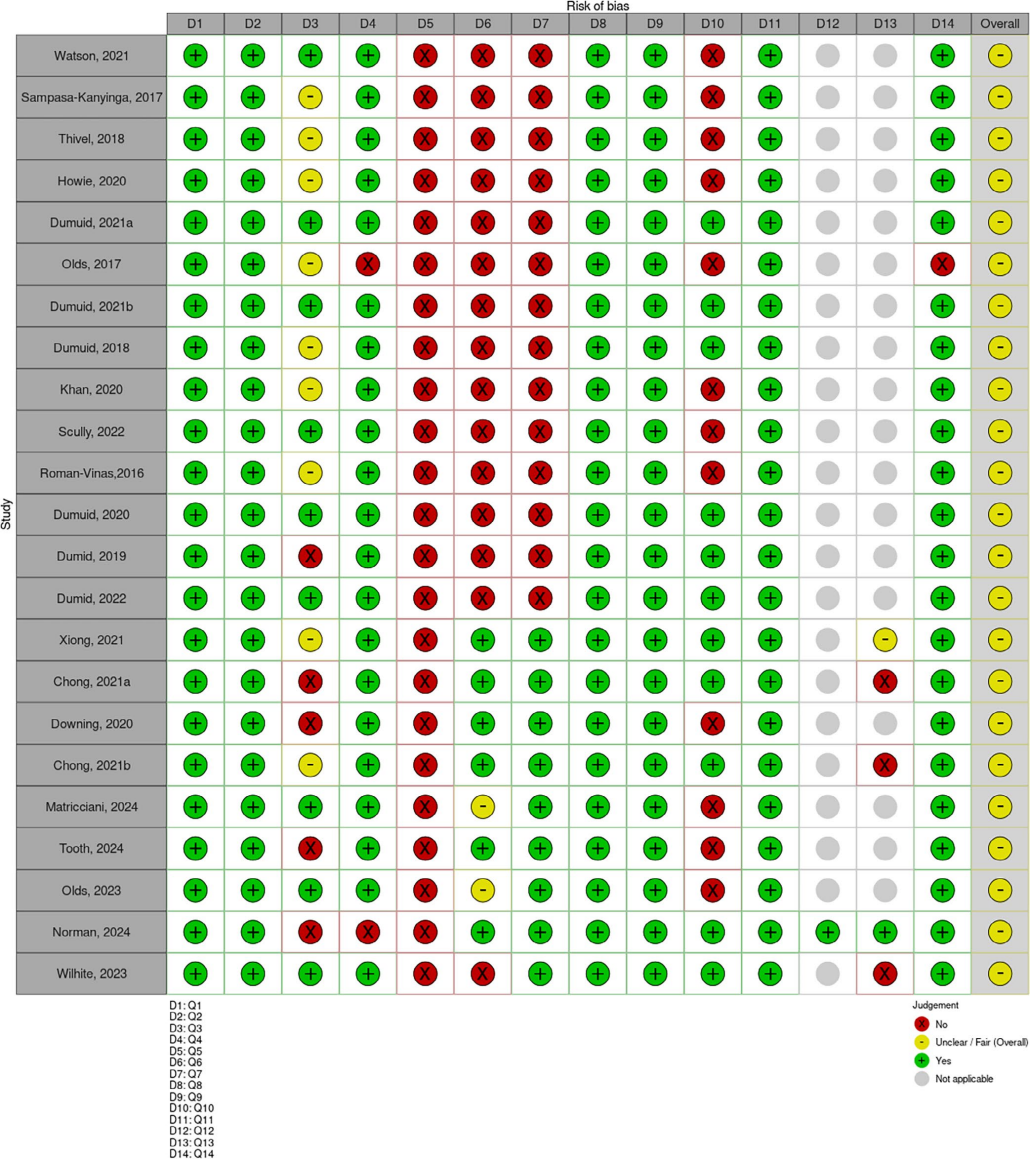
Quality of evidence in the included articles (*n* = 23).

The quality of the included articles was assessed using the National Institutes of Health Quality Assessment Tool [50]. These tools provide a comprehensive checklist tailored to a specific study type (observational or interventional). For observational studies, the criteria included clarity of research questions, specification of the study population, assessment rates, justification of sample size and consistent and valid measurement of exposure and outcomes. For interventional studies, the checklist covered aspects such as adequacy of randomisation, concealment of treatment allocation, blinding of participants and outcome assessors, similarity of groups at baseline, adherence to intervention protocols and use of intention‐to‐treat analysis (Data S1). Each article was scored based on these criteria: studies with ≥ 80% (at least 12 out of 14 Yes scores) were considered good, those with 30%–80% (4–11 out of 14 Yes scores) were considered fair and those with < 30% (3 out of 14 Yes scores) were considered poor. All of the articles were of fair quality (Figure 2). This current review used publicly accessible data, eliminating the need for ethical approval.

## 3 Results

**FIGURE 3:**
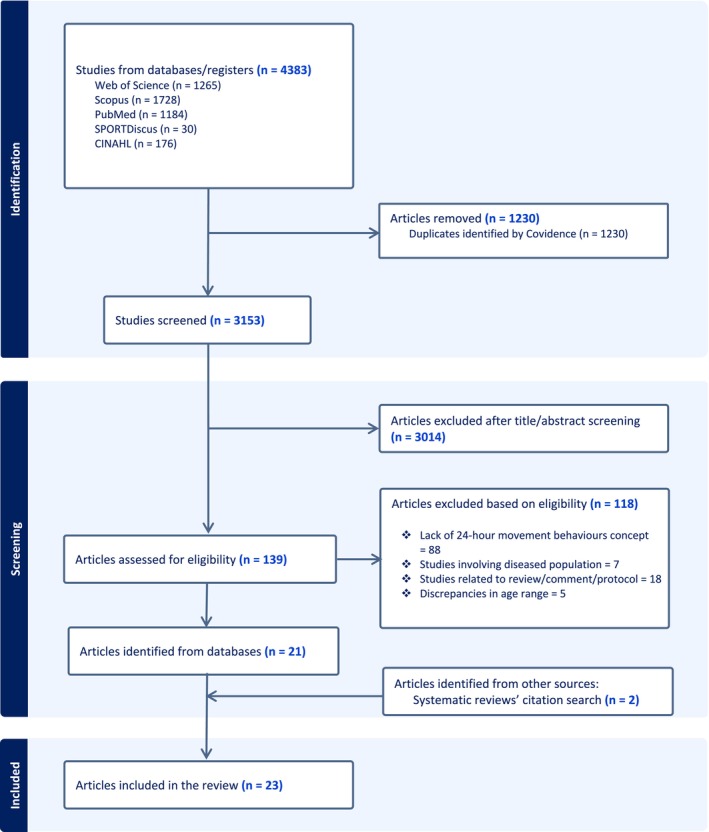
PRISMA flow diagram for article selection for the scoping review.

From 4383 articles screened, 23 were suitable for inclusion in the review (Figure 3). These covered themes such as the prevalence of meeting 24‐h MB guidelines (*n* = 12) [7, 8, 21, 22, 23, 24, 25, 26, 27, 31, 32, 34], correlates (*n* = 5) [23, 24, 32, 39, 51], health and well‐being outcomes (*n* = 13) [7, 8, 25, 26, 27, 28, 29, 30, 35, 36, 37, 38, 52], academic performance (*n* = 4) [21, 22, 28, 29] and interventions (*n* = 1) [33]. Outcomes included enhanced academic performance (*n* = 4) [21, 22, 28, 29], higher quality of life (*n* = 5) [7, 8, 27, 29, 30] and improved physical (*n* = 8) [25, 26, 28, 29, 35, 36, 37, 38] and mental (*n* = 2) [28, 29] health. Additionally, the intervention study indicated that parental text messaging was a promising strategy in promoting healthy behaviours, particularly in increasing PA in children (*n* = 1) [33].

### 3.1 24‐Hour Movement Behaviours Guidelines

Articles published using a 24‐h MB guidelines framework, mainly cross‐sectional [8, 21, 22, 23, 25, 26, 27, 31, 32] and longitudinal [7, 24, 39], were conducted across Australia. These studies involved sample sizes ranging from 182 to 33 201 children, with a mean age between 7.2 and 14.7 years [7, 8, 21, 22, 23, 24, 25, 26, 27, 31, 32, 33, 39]. The measurement tools included accelerometers [26, 27] and child‐reported and parent‐reported questionnaires [7, 8, 21, 22, 23, 24, 25, 31, 32, 33, 39]. Most articles relied on self‐reported data, with only a few articles reported findings using objective measurements [22, 25, 26, 27] (Table S1).

The compliance with the guidelines for three behaviours was low, ranging from 2% to 22.1% [7, 8, 21, 22, 23, 24, 25, 26, 27, 31, 32, 34], mostly below 10% [8, 21, 23, 25, 31, 32]. Boys typically showed higher compliance than girls, while lower socioeconomic status and having a family history of diabetes was associated with lower compliance [7, 23, 24, 39]. Moreover, a healthier dietary pattern was associated with greater adherence to guidelines [25]. Regarding outcomes, children who met all three behaviour guidelines consistently had better academic performance [21, 22], particularly in literacy and numeracy [21]. They also demonstrated improved health outcomes, including lower BMI, a reduced risk of obesity [25, 26, 28, 29, 35, 36, 37, 38] and higher health‐related quality of life (HRQoL) scores [7, 8, 27, 29, 30]. However, articles examining diverse aspects of health and developmental outcomes were limited along with a lack of causal link with the outcomes and associated factors. A pilot study on parental text message interventions reported that 77% of participants felt that these messages positively influenced their children's movement behaviours, with high approval for both the interaction and the frequency of messages, although these findings are limited, as they are based on a single study [33].

Some of the included studies showed non‐significant associations between the variables of interest. No statistically significant differences were observed in literacy or numeracy achievement based on the number of guidelines met, where the behaviours were assessed using accelerometry [22]. Another study reported that the proportion of students meeting all three key behavioural guidelines did not vary significantly by sex, grade level, or socioeconomic area [23]. Additionally, no associations were observed between a family history of heart disease and the odds of meeting the guidelines [24].

### 3.2 24‐Hour Movement Composition

The reviewed articles used cross‐sectional [28, 29, 30, 35, 36, 37, 38, 51] and longitudinal designs [34, 52] in various Australian settings, with sample sizes ranging from 83 to 4526 participants [28, 29, 30, 34, 35, 36, 37, 38, 51, 52]. Data collection methods varied, with eight articles utilising device‐measured data [28, 30, 34, 35, 36, 37, 38, 52], while two of the articles relied entirely on self‐reported data [29, 51]. The statistical technique used was compositional regression analysis (compositional multivariate and isometric log‐ratio mixed‐effect linear models) [28, 29, 30, 34, 35, 36, 37, 38, 52] (Table S2).

Reallocating time between different movement behaviours, which can be categorised as one‐to‐one, one‐to‐many, many‐to‐one, and many‐to‐many behaviours, yielded varied outcomes (Table 1). Specifically, reallocating time to LPA from sleep, SB, and MVPA was linked to increased internalising problems and total difficulty scores [52] while also being associated with changes in sleep patterns [38]. Conversely, reallocating time to SB from MVPA, LPA and sleep had positive association with internalising problems [52], sleep onset, efficiency and consistency but was negatively associated with sleep offset [38]. Increasing time spent sleeping by reallocating from LPA, MVPA and SB was negatively associated with internalising problems, total difficulty scores, sleep onset, sleep variability and efficiency [38, 52], while positively associated with sleep offset [38]. Shifting time to MVPA from LPA, SB and sleep was linked to lower body fat percentage, improved fat‐free mass, and enhanced HRQoL [30, 35]. However, this shift was negatively associated with sleep offset [38].

**TABLE 1:** Health and developmental outcomes associated with reallocations of time between sleep, SB, LPA and MVPA.

	Composition								
Study details	Sleep	MVPA	LPA	SB	Outcome				
Dumuid et al. [30]	**↓**	**↑**	**↓**	**↓**	**↑** HRQOL				
Dumid et al. [35]	**↑**	**↑**	**↑**	**↓**	**↔**Truncal fat	**↓** Nontruncal fat		**↑** Fat‐free mass	
	**↑**	**↑**	**↓**	**↑**	**↓**Truncal fat	**↓** Nontruncal fat		**↑** Fat‐free mass	
	**↑**	**↓**	**↑**	**↑**	**↑**Truncal fat	**↑** Nontruncal fat		**↓** Fat‐free mass	
	**↓**	**↓**	**↓**	**↑**	**↔**Truncal fat	**↑** Nontruncal fat		**↓** Fat‐free mass	
	**↓**	**↓**	**↑**	**↓**	**↑**Truncal fat	**↑** Nontruncal fat		**↓** Fat‐free mass	
	**↓**	**↑**	**↓**	**↓**	**↓**Truncal fat	**↓** Nontruncal fat		**↑** Fat‐free mass	
	**↓**	**↔**	**↔**	**↑**	**↔**Truncal fat	**↑** Nontruncal fat		**↓** Fat‐free mass	
	**↓**	**↔**	**↑**	**↔**	**↑**Truncal fat	**↑** Nontruncal fat		**↓** Fat‐free mass	
	**↓**	**↑**	**↔**	**↔**	**↓**Truncal fat	**↓** Nontruncal fat		**↑** Fat‐free mass	
	**↑**	**↔**	**↔**	**↓**	**↔**Truncal fat	**↓** Nontruncal fat		**↔** Fat‐free mass	
	**↔**	**↔**	**↑**	**↓**	**↑**Truncal fat	**↔** Nontruncal fat		**↓**Fat‐free mass	
	**↔**	**↑**	**↔**	**↓**	**↓**Truncal fat	**↓**Nontruncal fat		**↑**Fat‐free mass	
	**↑**	**↔**	**↓**	**↔**	**↓**Truncal fat	**↓**Nontruncal fat		**↑**Fat‐free mass	
	**↔**	**↔**	**↓**	**↑**	**↓**Truncal fat	**↔**Nontruncal fat		**↑**Fat‐free mass	
	**↔**	**↑**	**↓**	**↔**	**↓**Truncal fat	**↓** Nontruncal fat		**↑**Fat‐free mass	
	**↑**	**↓**	**↔**	**↔**	**↑** Truncal fat	**↑** Nontruncal fat		**↓** Fat‐free mass	
	**↔**	**↓**	**↔**	**↑**	**↑** Truncal fat	**↑** Nontruncal fat		**↓** Fat‐free mass	
	**↔**	**↓**	**↑**	**↔**	**↑** Truncal fat	**↑** Nontruncal fat		**↓** Fat‐free mass	
Dumuid et al. [36]	**↑**	**↑**	**↑**	**↓**	**↑** Trabecular density		**↑** Endosteal circumference		
	**↓**	**↑**	**↓**	**↑**	**↑** Cortical density				
	**↑**	**↑**	**↓**	**↓**	**↑** Periosteal circumference	**↑** Polar moment of inertia		**↑** Polar SSI	
Dumuid et al. [37]	**↓**	**↑**	**↑**	**↓**	**↑** Standing broad jump	**↑** VO2max		**↑** Fitness composite	
	**↑**	**↑**	**↓**	**↓**	**↑** BMIz	**↑** Waist‐to‐height ratio	**↑** Fat‐to‐fat‐free mass	**↑** Adiposity composite	
Dumuid et al. [28]	**↑**	**↑**	**↓**	**↓**	**↑** Mental health		**↑** Physical health		
	**↑**	**↓**	**↓**	**↑**	**↑** Cognitive/academic				
	**↑**	**↑**	**↓**	**↑**	**↑** Overall health and well‐being (equal)	**↑** Overall health and well‐being (prioritise mental health)	**↑** Overall health and well‐being (prioritise cognitive health/academic)	**↑** Overall health and well‐being (prioritise physical health)	
Dumid et al. [29]	↔	**↑**	↔	**↓**	**↓** Body fat	**↑** Psychosocial health		**↑** Academic performance	
	**↓**	**↑**	**↓**	**↓**	**↓** Body fat	**↑** Psychosocial health		**↓** Academic performance	
Matricciani et al. [38]	**↑**	**↓**	**↓**	**↓**	**↓** Sleep efficiency	**↓** Sleep onset	**↑** Sleep offset	**↓** Sleep variability	
	**↓**	**↑**	**↓**	**↓**	↔ Sleep efficiency	↔ Sleep onset	**↓** Sleep offset	↔ Sleep variability	
	**↓**	**↓**	**↓**	**↑**	**↑** Sleep efficiency	**↑** Sleep onset	**↓** Sleep offset	**↑** Sleep variability	
	**↓**	**↓**	**↑**	**↓**	↔ Sleep efficiency	**↑** Sleep onset	**↓** Sleep offset	**↑** Sleep variability	
Chong et al. Longitudinal^a^ and Cross‐sectional [52]	**↑**	**↓**	**↓**	**↓**	**↓** Internalising problems	↔ Externalising problems	**↓** Total difficulties	↔ Prosocial behaviour	↔ Psychological distress
	**↓**	**↓**	**↓**	**↑**	**↑** Internalising problems	↔ Externalising problems	↔ Total difficulties	↔ Prosocial behaviour	↔ Psychological distress
	**↓**	**↓**	**↑**	**↓**	**↑** Internalising problems	↔ Externalising problems	**↑** Total difficulties	↔ Prosocial behaviour	↔ Psychological distress
	**↓**	**↑**	**↓**	**↓**	↔ Internalising problems	↔ Externalising problems	↔ Total difficulties	↔ Prosocial behaviour	↔ Psychological distress

*Note:* ↑: Significant Increase, ↓: Significant Decrease, ↔: No Significant Change.

^a^ Longitudinal reallocations were not significantly associated with health and developmental outcomes. The study by Chong et al. (2021) [34] was not included in the summary as it focused on longitudinal changes in 24‐h movement composition rather than specific outcomes. Similarly, the study by Olds et al. (2023) [51] was excluded as it primarily characterised time spent in behaviours across individuals from different social backgrounds.

Furthermore, reallocating time from SB to MVPA was associated with improved psychosocial health and academic performance, while reducing body fat percentage [29]. Similarly, reallocating time from SB to other behaviours was linked to increased trabecular density and endosteal circumference [37]. The optimal time‐use composition for holistic health includes reducing LPA and reallocating time to MVPA, SB and sleep [28]. For optimal skeletal health, the “Goldilocks” daily time‐use composition for 11–12 years children includes around 10.9 h of sleep, 8.2 h of sedentary time, 3.4 h of LPA and 1.5 h of MVPA [36]. For fitness and adiposity, the optimal composition includes 10.2 h of sleep, 9.9 h of sedentary time, 2.4 h of LPA and 1.5 h of MVPA [37]. On average, the ideal “Goldilocks” day comprises 10 h 21 min of sleep, 9 h 44 min of sedentary time, 2 h 26 min of LPA and 1 h 29 min of MVPA [28].

During the transition from primary to secondary school, compliance with 24‐h MB guidelines declined significantly [34]. These changes in movement behaviours were particularly pronounced on weekdays, with an increase in recreational screen time and out‐of‐school educational activities [34]. However, longitudinal studies examining this transitional period, along with compliance patterns at the onset of childhood and adolescence, are limited.

## 4 Discussion

This systematic scoping review highlights that the current evidence on 24‐h movement behaviour (24‐h MB) guidelines among Australian children and adolescents is still developing, with only 23 studies published over the past 8 years. This limited body of research constrains our understanding of adherence levels and the factors influencing these behaviours. Despite the existence of these guidelines, compliance remains critically low, with reported adherence ranging from 2% to 22% across studies. This indicates a substantial disconnect between the guidelines and actual behaviours, underscoring the need for more effective strategies to promote adherence. The existing literature has explored and found associations between adherence and factors such as socioeconomic status, sex and family health history, as well as outcomes such as physical health, cognitive development, emotional well‐being and academic performance. However, while some findings show associations between adherence to guidelines and improved health and developmental outcomes, some studies reported no significant relationships. For instance, no significant differences were found in numeracy or literacy achievement when guidelines compliance was assessed using an accelerometer, nor were adherence rates by sex, grade level, socioeconomic area or association with a family history of heart disease. Moreover, these findings are often narrow in scope, focusing on specific health or developmental outcomes without considering a holistic approach and are limited by the predominance of cross‐sectional studies and reliance on subjective or self‐reported measures, which restricts the ability to draw causal inferences and fully understand the determinants of adherence. Also, intervention studies are notably scarce, with only one pilot randomised controlled trial identified in this review, highlighting a significant gap in the development and evaluation of strategies to improve adherence. The inclusion of compositional data analysis (CoDA) studies adds a nuanced perspective by examining how reallocation of time among different movement behaviours is associated with various health outcomes, emphasising the importance of a balanced daily routine. These synthesised insights contribute to the literature by identifying existing gaps and suggesting areas where future research and targeted interventions might help enhance adherence to the 24‐h MB guidelines among Australian children and adolescents.

The low adherence rates to 24‐h MB guidelines [8, 21, 23, 25, 31, 32] among Australian children and adolescents may indicate that current health promotion strategies are insufficient and that expecting compliance with the three simultaneous guidelines presents inherent challenges. Balancing PA, ST and sleep may create cognitive demands and conflicting priorities, making it difficult for youth to effectively maintain all three behaviours. Additionally, factors such as limited access to safe play areas [53] may hinder PA opportunities, while the increasing prevalence of ST‐promoting activities (e.g., gaming and social media) further exacerbates the difficulty of adhering to guidelines, as excessive ST directly contradicts the recommendations of the 24‐h MB framework [54]. Socioeconomic disparities can exacerbate these issues, with children from lower‐income families facing greater barriers to PA owing to limited resources and opportunities [53]. Additionally, increased ST and academic pressures are linked to shorter sleep durations among Australian children [55]. Addressing these environmental and social determinants is crucial for improving the adherence rates.

Lower socioeconomic status, girls and family health history of diabetes were significantly negatively associated with adherence to 24‐h MB guidelines [7, 23, 24, 39]. The possible reasons behind these patterns could be explained in several ways. For example, a higher socioeconomic status often correlates with better access to PA resources, such as sports facilities and recreational programs compared to lower [56], while sex differences may reflect varying social expectations and opportunities for PA [57]. Mechanistically, energy expenditure patterns and motor activity tendencies during adolescence may contribute to differences in PA. Boys often experience greater increases in lean muscle mass during puberty [58], which can lead to higher energy expenditure during activities and encourage participation in vigorous movements. Girls, however, may experience relatively lower increases in lean muscle mass and a shift toward fat mass [58], which may influence their preference for less vigorous activities. Moreover, in Australia, boys typically have more opportunities for active play than girls, who may face more restrictions and societal expectations [59]. Furthermore, children from families with active lifestyles or health‐conscious behaviours are more likely to meet guidelines because of positive role modelling and support [60, 61, 62]. However, the absence of significant differences in adherence rates by sex, grade level and socioeconomic area [23] or associations with a family history of heart disease in some studies suggests that other unexplored factors or variations in measurement methods may influence these findings [24]. These mixed findings underscore the importance of further research and targeted interventions that consider both demographic and familial factors.

The positive health outcomes associated with adherence to these guidelines are compelling. Lower BMI, lower likelihood of obesity, healthier dietary patterns and higher HRQoL scores among compliant children highlight the comprehensive benefits of following guidelines, as evidenced in Australian studies [25, 26, 28, 29, 35, 36, 37, 38]. Possible mechanisms involve interplay between PA, SB, and sleep. Increased PA is associated with better energy balance [63] and metabolic health [64], whereas structured daily routines that include adequate sleep may support overall recovery and cognitive function [65]. Additionally, reducing ST is essential as it encourages more PA [66] and allows sufficient restorative sleep, which is important for maintaining a healthier lifestyle and promoting overall health [67]. This integrated approach underscores the importance of managing these behaviours collectively rather than in isolation. It is also important to note that HRQoL measures often incorporate assessments of PA, which means that the higher HRQoL scores may partly reflect increased PA levels among compliant children. Tailored strategies to increase adherence with all three behaviours together, such as school‐based programs, community initiatives and policy changes that create supportive environments for healthy behaviours, are crucial in bridging the gap in health outcomes.

The correlation between compliance with 24‐h MB and improved academic performance, particularly in literacy and numeracy, has been observed in Australian studies [21, 22]. This correlation can be attributed to several mechanisms: PA may enhance cognitive function through increased blood flow and oxygen supply, which supports learning and memory [68]. Adequate sleep is essential for cognitive processes, including attention and memory consolidation [69], while limited ST may reduce cognitive overload and distractions [9]. However, the relationship between ST and cognitive outcomes is complex and sometimes contradictory across studies. While excessive passive ST (such as prolonged TV watching) is generally associated with poorer cognitive outcomes, certain types of ST – such as educational games, interactive learning platforms, or moderated use of social tools – are positively linked to improvements in cognitive skills, including problem‐solving and critical thinking [70]. Some researchers have even suggested a connection between ST and the Flynn Effect, which refers to the observed rise in average IQ scores over time potentially linked to increased exposure to complex digital environments [71]. These variations in findings may be attributed to differences in the purpose, duration and context of ST, as well as differences in study design and the cognitive outcomes measured. This dual role of ST, combined with the critical roles of PA and sleep in supporting cognitive function, highlights the intricate ways in which these behaviours may be collectively associated with academic performance. While these observations are specific to the Australian context, they align with broader global findings that suggest similar patterns [40].

Given the pivotal role of ST in academic and cognitive outcomes, its inclusion in the broader SB recommendations likely reflects its prominence in the daily lives of children and adolescents [72]. However, equating ST with SB carries significant implications and risks. By treating ST as synonymous with SB, we may overlook other substantial non‐screen‐based sedentary activities such as reading, homework, or sitting during transportation [73]. This oversimplification can lead to incomplete assessments of a child's overall sedentary lifestyle, potentially underestimating the true extent of SB [74]. Consequently, interventions that focus on reducing ST might miss critical areas where excessive sedentariness occurs, thereby limiting the effectiveness of strategies aimed at promoting healthier and more active movement behaviours. Recognising the multifaceted nature of SB is essential for developing comprehensive guidelines that address all sources of sedentariness, ensuring a more accurate and holistic approach for improving the health and well‐being of children and adolescents.

Preliminary results from a pilot study indicate that parental text‐messaging interventions may be effective in promoting adherence to 24‐h MB guidelines, though further research is needed to confirm these findings [33]. In Australia, where mobile phone usage is widespread, such parental interventions are scalable and cost effective [75]. High acceptance and reported behavioural changes suggest that these digital strategies may effectively encourage healthier behaviours [76, 77]. However, the long‐term effectiveness of these parental interventions warrants further research to ensure sustained behavioural changes [76]. Integrating these interventions with broader public health strategies could enhance their impact and reach, making them more effective in promoting adherence to the movement guidelines.

Compositional analyses highlight the importance of balancing PA, SB and sleep for holistic health. Reallocation of time to MVPA from sleep, LPA and SB is associated with improvements in body composition and psychosocial health [30, 35]. This improvement is achieved through mechanisms such as spending more time for MVPA increased metabolic rate, improved cardiovascular health [78] and the release of endorphins, which elevate mood and reduce stress [79]. Similarly, reallocating time to sleep from LPA, MVPA and SB is associated with better mental health outcomes because adequate sleep is crucial for cognitive function, emotional regulation, and overall psychological well‐being [38, 52]. In Australia, where SB such as ST are prevalent [80], promoting an optimal mix of activities is crucial. Interventions should encourage a balanced daily routine, integrating sufficient PA, while reducing SB. This balanced approach can help mitigate the negative effects of excessive SB and inadequate PA, providing comprehensive health benefits.

The transition from primary to secondary school represents a critical period with notable changes in movement behaviours. Increased academic demands and social adjustments during the transition from primary to secondary school can lead to shorter sleep durations [55]. Coupled with reduced opportunities for PA [81], these factors often result in decreased adherence to movement behaviours guidelines. In the Australian school system, this transition can involve more structured and sedentary classroom environments [82]. Addressing these challenges through targeted interventions such as structured educational programmes and supportive school policies is essential. Ensuring continuity and support during this transitional phase can help maintain and improve adherence to movement guidelines and promote better long‐term health outcomes in adolescents.

The predominance of cross‐sectional studies and reliance on subjective reporting in the literature may be attributed to practical and logistical challenges. Conducting longitudinal and experimental research requires substantial time, funding, and participant commitment, which can be difficult to achieve [83, 84]. Subjective measures are often more feasible and cost‐effective than objective assessments such as accelerometers or wearable devices, especially in large populations of children and adolescents [85]. However, subjective measures can yield different adherence estimates compared with objective methods because of differences in how behaviours are recorded. For instance, accelerometer‐measured sleep durations are typically longer than self‐reported durations in some studies [86] because accelerometers interpret immobility as sleep, which may mistakenly include periods of wakefulness [87]. Conversely, self‐reports may underestimate sleep due to recall bias or misjudgment of the total time spent asleep. Similarly, accelerometer‐measured MVPA often produces lower estimates than self‐reported MVPA because accelerometers rely on movement thresholds and may not capture activities such as cycling or swimming [88]. However, accelerometers remain a valuable tool for objectively quantifying movement patterns, providing data that can improve the accuracy of adherence estimates, though potential biases may still arise depending on placement, wear time, and data interpretation. In a study of Australian children and adolescents, adherence to all three 24‐h MB guidelines was reported to be 20.3% via self‐reports and 12.0% via accelerometry [22]. With these, in the same study, while self‐reported measures indicated better numeracy and literacy achievement based on the number of guidelines met, accelerometer‐derived measures showed no significant differences [22]. These discrepancies underscore the need for standardised measurement approaches – such as harmonising accelerometer protocols, data thresholds, and interpretation criteria – to improve the comparability and reliability of adherence estimates in 24‐h MB research, as they may lead to inconsistent interpretations and impact the development of evidence‐based interventions and policies. On the other hand, the reliance on cross‐sectional design limits the ability to establish causality and fully understand the determinants of adherence to 24‐h MB guidelines. The lack of intervention studies, with only one pilot randomised controlled trial identified, underscores the significant gap in efforts to improve adherence. It is crucial to prioritise research that utilises longitudinal and experimental designs with objective measurement tools. Such an approach would enhance the accuracy of the findings, allow for causal inferences, and inform the development of effective interventions. Addressing these methodological limitations is essential for advancing knowledge in this field and ultimately for improving health outcomes among Australian children and adolescents.

Positioning the current findings within the global literature on 24‐h MB reveals that the challenges observed in Australia align closely with broader global trends. Low adherence to the 24‐h MB guidelines is a worldwide issue, with only 7.12% of youths from 23 countries meeting all three recommendations, particularly among adolescents and girls [42]. Similar to the global situation [10, 42], research efforts in Australia have been constrained by reliance on cross‐sectional studies, which limits their ability to draw causal inferences. Although Australia has made progress by conducting some longitudinal studies to better understand adherence and its outcomes, such studies remain scarce, both locally and internationally [10, 42]. Intervention studies aiming to improve adherence to the 24‐h MB guidelines are also still limited. Promising community‐based and school‐driven initiatives from Canada [89, 90] and Spain [91, 92] have emerged; however, these efforts are not widespread, revealing a significant gap in effective intervention development on a global scale. This gap presents an opportunity for Australia to adapt and expand on these existing strategies, while contributing to a broader understanding of how best to enhance adherence to 24‐h MBs, and ultimately bridge the gaps in health and developmental outcomes among children and adolescents.

## 5 Limitations and Future Directions

This scoping review has several limitations. First, there may be a selection bias due to variations in the selection of study populations among the included studies, which could affect the generalisability of our findings. Second, reporting bias was a concern because many of the included studies relied on self‐reported data, potentially affecting the accuracy of the synthesised results. However, the quality of the articles was evaluated using the NIH Quality Assessment Tools, focusing on criteria such as study population selection, consistency of outcome measures, and data collection methods. Furthermore, the predominance of cross‐sectional studies among the included literature limits the ability of this review to explore temporal relationships between 24‐h movement behaviours and health outcomes, as these studies capture data at a single point in time. Although a few articles with longitudinal and experimental designs were included, they did not provide sufficient evidence to understand causal changes over time, which constrains our ability to draw stronger conclusions within the context of this review. Additionally, there is inconsistency in the conceptualisation of sedentary behaviour across the included articles. Some articles focused on the 24‐h movement behaviour guidelines, which include screen time as an indicator of sedentary behaviour, while others examined sedentary behaviour more broadly within 24‐h movement compositions. This discrepancy has led to challenges in comparing and synthesising findings across articles. Variability in the measurement tools and study designs further complicates direct comparisons and may affect the conclusions drawn from this review. These limitations suggest that future research should address these gaps by conducting well‐designed longitudinal and experimental studies to better understand the relationship between movement behaviours and outcomes, such as health and academic performance over time.

## 6 Conclusions

Although there has been some research on 24‐h movement behaviours (24‐h MB) among Australian children and adolescents, the body of evidence remains limited, with only 23 studies published over the past 8 years since the integrated guidelines were first introduced. Adherence to these guidelines remains low, reflecting challenges such as limited research on effective intervention strategies, insufficient school and community engagement to support balanced movement behaviours, disparities in physical activity and sleep among different socioeconomic groups and increasing screen activities. This systematic review identified critical gaps in understanding the factors contributing to noncompliance and the potential relationships between 24‐h MBs and various health and academic outcomes, especially causal relationships. Additionally, the predominance of self‐reported data limits the reliability of current findings, emphasising the need for more device‐based measures to improve the accuracy of findings. Future research should prioritise longitudinal studies to establish these causal links and explore the socioeconomic and cultural barriers that hinder adherence. Additionally, there is a pressing need to develop and evaluate scalable and innovative interventions, such as digital health strategies, that can effectively promote sustained behavioural changes. Addressing these gaps can lay the groundwork for more tailored public health policies and interventions aimed at supporting the health and well‐being of Australian youth at crucial developmental stages.

## Author Contributions

**Mosharop Hossian:** conceptualization, methodology, data curation, software, and original draft preparation. **Mehwish Nisar:** data curation, visualization, investigation, and writing – reviewing and editing. **Gregore Iven Mielke:** conceptualization, validation, and writing – reviewing and editing. **Asaduzzaman Khan:** conceptualization, methodology, writing – reviewing and editing, and supervision.

## Conflicts of Interest

The authors declare no conflicts of interest.

## Supporting information


Data S1.

**Table S1.** Summary of the key characteristics of the included articles using 24‐h movement behaviours framework.

**Table S2.** Summary of the key characteristics of the included articles using 24‐h movement composition.

Data S2.

## Data Availability

The authors confirm that the data supporting the findings of this study are available within the article and its Supporting Informations.
