# Supplementary material for: Twenty Four‐Hour Movement Behaviours Research Among Australian Children and Adolescents: A Scoping Review
Source: Health Promot J Austr. 2025 Feb 19;36(2):e70021. doi: 10.1002/hpja.70021 (PMC11836639; doi:10.1002/hpja.70021)
Supplement: Supplementary file 4 — Data S2. [file HPJA-36-0-s003.docx]

**Table 1.** Search strategy in different databases (Primary).

| **Data Base** | **Search strategy** | **Filters** |
| --- | --- | --- |
| Pubmed  (*n* = 1110) | (((((((((exercise[Title/Abstract]) OR (exercise[Mesh])) OR ("physical activit*"[Title/Abstract]))) OR ("sedentary behavior" [Mesh] OR "screen time" [Mesh] OR sedentar*[Title/Abstract] OR inactiv*[Title/Abstract] OR "screen time"[Title/Abstract] OR "screen based"[Title/Abstract] )) OR ((sleep [Mesh]) OR (sleep*[Title/Abstract]))) OR ("movement*"[Title/Abstract] OR 24-h*[Title/Abstract])) AND (guideline*[Title/Abstract] OR recommendation*[Title/Abstract])) AND (child*[Title/Abstract] OR adolescen*[Title/Abstract] OR young*[Title/Abstract] OR youth*[Title/Abstract] OR student*[Title/Abstract] OR Adolescents [Mesh] OR Child [Mesh])) AND (Australia) | Since: 2016 |
| Scopus  (*n* = 1613) | ( ( ( ( ( ( ( ( ( TITLE-ABS ( exercise ) ) OR ( INDEXTERMS ( exercise ) ) ) OR ( TITLE-ABS ( "physical activit*" ) ) ) ) OR ( INDEXTERMS ( "sedentary behavior" ) OR INDEXTERMS ( "screen time" ) OR TITLE-ABS ( sedentar* ) OR TITLE-ABS ( inactiv* ) OR TITLE-ABS ( "screen time" ) OR TITLE-ABS ( "screen based" ) ) ) OR ( ( INDEXTERMS ( sleep ) ) OR ( TITLE-ABS ( sleep* ) ) ) ) OR ( TITLE-ABS ( movement* ) OR TITLE-ABS ( 24-h* ) ) ) AND ( TITLE-ABS ( guideline* ) OR TITLE-ABS ( recommendation* ) ) ) AND ( TITLE-ABS ( child* ) OR TITLE-ABS ( adolescen* ) OR TITLE-ABS ( young* ) OR TITLE-ABS ( youth* ) OR TITLE-ABS ( student* ) OR INDEXTERMS ( adolescents ) OR INDEXTERMS ( child ) ) ) AND ( australia ) | Since: 2016;  Language: English;  Document type: article |
| Web of Science  (*n* = 1165) | ((((((((((TI=exercise OR AB=exercise)) OR (ALL=exercise)) OR ((TI="physical activit*" OR AB="physical activit*")))) OR (ALL="sedentary behavior" OR ALL="screen time" OR (TI=sedentar* OR AB=sedentar*) OR (TI=inactiv* OR AB=inactiv*) OR (TI="screen time" OR AB="screen time") OR (TI="screen based" OR AB="screen based"))) OR ((ALL=sleep) OR ((TI=sleep* OR AB=sleep*)))) OR ((TI=movement* OR AB=movement*) OR (TI=24-h* OR AB=24-h*))) AND ((TI=guideline* OR AB=guideline*) OR (TI=recommendation* OR AB=recommendation*))) AND ((TI=child* OR AB=child*) OR (TI=adolescen* OR AB=adolescen*) OR (TI=young* OR AB=young*) OR (TI=youth* OR AB=youth*) OR (TI=student* OR AB=student*) OR ALL=Adolescents OR ALL=Child)) AND ((ALL = Australia)) | Since: 2016;  Language: English;  Document types: article |
| SportDiscus (n = 30) | ((((((((((TI "exercise" OR AB "exercise")) OR (DE "exercise")) OR ((TI "physical activit*" OR AB "physical activit*")))) OR (DE "sedentary behavior" OR DE "screen time" OR (TI "sedentar*" OR AB "sedentar*") OR (TI "inactiv*" OR AB "inactiv*") OR (TI "screen time" OR AB "screen time") OR (TI "screen based" OR AB "screen based"))) OR ((DE "sleep") OR ((TI "sleep*" OR AB "sleep*")))) OR ((TI "movement*" OR AB "movement*") OR (TI "24-h*" OR AB "24-h*"))) AND ((TI "guideline*" OR AB "guideline*") OR (TI "recommendation*" OR AB "recommendation*"))) AND ((TI "child*" OR AB "child*") OR (TI "adolescen*" OR AB "adolescen*") OR (TI "young*" OR AB "young*") OR (TI "youth*" OR AB "youth*") OR (TI "student*" OR AB "student*") OR DE "Adolescents" OR DE "Child")) AND ((DE "Australia")) | Since: 2016;  Language: English; |
| CINAHL (n = 176) | ((((((((((TI exercise OR AB exercise)) OR ((MH exercise+))) OR ((TI "physical activit*" OR AB "physical activit*")))) OR ((MH "sedentary behavior+") OR (MH "screen time+") OR (TI sedentar* OR AB sedentar*) OR (TI inactiv* OR AB inactiv*) OR (TI "screen time" OR AB "screen time") OR (TI "screen based" OR AB "screen based"))) OR (((MH sleep+)) OR ((TI sleep* OR AB sleep*)))) OR ((TI movement* OR AB movement*) OR (TI 24-h* OR AB 24-h*))) AND ((TI guideline* OR AB guideline*) OR (TI recommendation* OR AB recommendation*))) AND ((TI child* OR AB child*) OR (TI adolescen* OR AB adolescen*) OR (TI young* OR AB young*) OR (TI youth* OR AB youth*) OR (TI student* OR AB student*) OR (MH Adolescents+) OR (MH Child+))) AND ((MH Australia+)) | Since: 2016;  Language: English; |

**Table 2.** Search strategy in different databases (Compositional Data Analysis).

| **Data Base** | **Search strategy** | **Filters** |
| --- | --- | --- |
| Pubmed  (*n* = 74) | (("Compositional"[Title/Abstract]) AND ("24"[Title/Abstract])) AND (Australia) | Since: 2016 |
| Scopus  (*n* = 115) | ((TITLE-ABS(Compositional)) AND (TITLE-ABS(24))) AND (TITLE-ABS(Australia)) | Since: 2016;  Language: English;  Document type: article |
| Web of Science  (*n* = 100) | (((TI=Compositional OR AB=Compositional)) AND ((TI=24 OR AB=24))) AND ((ALL=Australia)) | Since: 2016;  Language: English; |
| SportDiscus (n = 0) | (((TI "Compositional" OR AB "Compositional")) AND ((TI "24" OR AB "24"))) AND ((DE "Australia")) |  |
| CINAHL (n = 0) | (((TI Compositional OR AB Compositional)) AND ((TI 24 OR AB 24))) AND ((MH Australia+)) |  |
